# Supplementary material for: Identification of photoperiod-induced specific miRNAs in the adrenal glands of Sunite sheep (Ovis aries)
Source: Front Vet Sci. 2022 Jul 22;9:888207. doi: 10.3389/fvets.2022.888207 (PMC9354845; doi:10.3389/fvets.2022.888207)
Supplement: Supplementary file 1 [file Data_Sheet_1.ZIP › Supplementary file/miRDeep2 file.zip!/miRDeep2 file/Know_Structure/LP42A2/oar-mir-143.pdf]

miRBase precursor : oar-mir-143
Total read count : 530325
oar-miR-143 read count : 526517
remaining reads : 3808

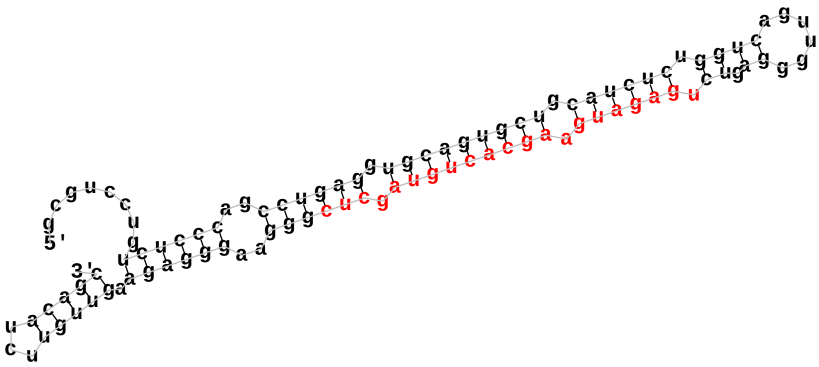

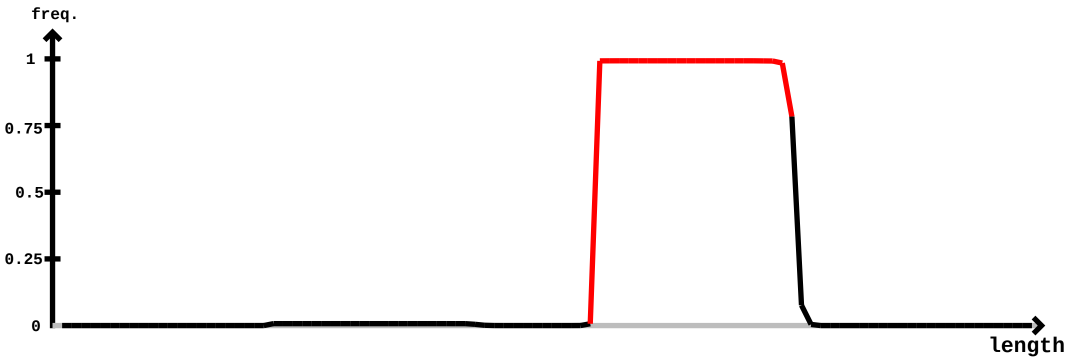


```
                                                  oar-miR-143
5'- gcguccugucucccagccugaggugcagugcugcaucucuggucaguugggagucugagaugaagcacuguagcucgggaagggagaaguuguucuacagc  -3'   exp
    ........((((((..((((((.(((((((((.((((((.((((......)).)).)))))).))))))))).))))))..)))))).(((((...)))))  reads  mm  sample
    ....................aggugcagugcugcaucucug............................................................  1      0   seq
    ....................aggugcagugcugcaucucugg...........................................................  2      0   seq
    ....................aggugcagugcugcaucucuggu..........................................................  2      0   seq
    ....................aggugcagugcugcaucucugguc.........................................................  1      0   seq
    .....................ggugcagugcugcaucucu.............................................................  13     0   seq
    .....................ggugcagugcugcaucucug............................................................  20     0   seq
    .....................ggugcagugcugcaucucugg...........................................................  1253   0   seq
    .....................ggugcagugcugcaucucuggu..........................................................  1909   0   seq
    .....................ggugcagugcugcaucucugguc.........................................................  535    0   seq
    .....................ggugcagugcugcaucucugguca........................................................  63     0   seq
    .....................ggugcagugcugcaucucuggucag.......................................................  1      0   seq
    ......................gugcagugcugcaucucuggu..........................................................  1      0   seq
    ......................gugcagugcugcaucucugguc.........................................................  2      0   seq
    .......................ugcagugcugcaucucugguca........................................................  1      0   seq
    ............................................aguugggagucugagaugaagcacu................................  1      0   seq
    ..............................................uugggagucugagaugaagcacu................................  1      0   seq
    ...................................................agucugagaugaagcacuguagc...........................  1      0   seq
    ....................................................gucugagaugaagcacuguagc...........................  1      0   seq
    .....................................................ucugagaugaagcacuguagc...........................  10     0   seq
    .....................................................ucugagaugaagcacuguagcu..........................  10     0   seq
    .....................................................ucugagaugaagcacuguagcuc.........................  84     0   seq
    ......................................................cugagaugaagcacugu..............................  3      0   seq
    ......................................................cugagaugaagcacugua.............................  1      0   seq
    ......................................................cugagaugaagcacuguag............................  2      0   seq
    ......................................................cugagaugaagcacuguagc...........................  75     0   seq
    ......................................................cugagaugaagcacuguagcu..........................  805    0   seq
    ......................................................cugagaugaagcacuguagcuc.........................  2541   0   seq
    ......................................................cugagaugaagcacuguagcucg........................  95     0   seq
    .......................................................ugagaugaagcacuguG.............................  3      1   seq
    .......................................................ugagaugGagcacugua.............................  1      1   seq
    .......................................................ugagGugaagcacugua.............................  3      1   seq
    .......................................................ugagaugaagcacuguU.............................  3      1   seq
    .......................................................ugagaugaagcacuCua.............................  1      1   seq
    .......................................................ugagaugaagcacugua.............................  202    0   seq
    .......................................................ugagaugaagcaUugua.............................  1      1   seq
```

oar-miR-143

```
gcguccugucucccagccugaggugcagugcugcaucucugguccaguugggagucugagaugaagcacuguagcucgggaagggagaaguuguucuacagc
.......................................................ugagaugaagUacugua.............................    4        1        seq
.......................................................ugagaugaagcacuguag............................    237      0        seq
.......................................................ugagaugaagcacuguagc...........................    3948     0        seq
.......................................................ugagaugaagcacuguagcu..........................    105553   0        seq
.......................................................ugagaugaagcacuguagcuc.........................    372470   0        seq
.......................................................ugagaugaagcacuguagcucg........................    38026    0        seq
.......................................................ugagaugaagcacuguagcucgg.......................    2242     0        seq
.......................................................ugagaugaagcacuguagcucggg......................    11       0        seq
........................................................gagaugaagcacuguagcu..........................    27       0        seq
........................................................gagaugaagcacuguagcuc.........................    95       0        seq
........................................................gagaugaagcacuguagcucg........................    16       0        seq
........................................................gagaugaagcacuguagcucgg.......................    1        0        seq
.........................................................agaugaagcacuguagcu..........................    2        0        seq
.........................................................agaugaagcacuguagcuc.........................    2        0        seq
.........................................................agaugaagcacuguagcucgg.......................    17       0        seq
.........................................................agaugaagcacuguagcucggg......................    4        0        seq
..........................................................gaugaagcacuguagcu..........................    1        0        seq
..........................................................gaugaagcacuguagcuc.........................    2        0        seq
..........................................................gaugaagcacuguagcucgg.......................    9        0        seq
..........................................................gaugaagcacuguagcucggg......................    1        0        seq
..........................................................gaugaagcacuguagcucggga.....................    3        0        seq
..........................................................gaugaagcacuguagcucgggaa....................    4        0        seq
...........................................................augaagcacuguagcuc.........................    1        0        seq
............................................................ugaagcacuguagcucgggaa....................    1        0        seq
```
